# Validation of the French translation-adaptation of the impact of cancer questionnaire version 2 (IOCv2) in a breast cancer survivor population

**DOI:** 10.1186/s12955-015-0301-x

**Published:** 2015-07-29

**Authors:** Myriam Blanchin, Sarah Dauchy, Alejandra Cano, Anne Brédart, Neil K. Aaronson, Jean-Benoit Hardouin

**Affiliations:** EA4275, Biostatistics, Pharmacoepidemiology and Subjective Measures in Health Sciences, University of Nantes, Nantes, France; Psycho-oncology Unit, Department of Supportive Care, Gustave Roussy, Villejuif, France; Psycho-oncology Unit, Department of Supportive Care – DISSPO, Institut Curie and University Paris Descartes, Psychopathology and Health Process Laboratory LPPS EA 4057, Paris, France; Division of Psychosocial Research and Epidemiology, The Netherlands Cancer Institute, Amsterdam, The Netherlands; Unit of Methodology and Biostatistics – University Hospital of Nantes, Nantes, France

**Keywords:** Cancer survivor, Survivorship, Breast cancer, Questionnaire, Psychometric properties, Psychosocial

## Abstract

**Background:**

The Impact of Cancer version 2 (IOCv2) was designed to assess the physical and psychosocial health experience of cancer survivors through its positive and negative impacts. Although the IOCv2 is available in English and Dutch, it has not yet been validated for use in French-speaking populations. The current study was undertaken to provide a comprehensive assessment of the reliability and validity of the French language version of the IOCv2 in a sample of breast cancer survivors.

**Methods:**

An adapted French version of the IOCv2 as well as demographic and medical information were completed by 243 women to validate the factor structure divergent/divergent validities and reliability. Concurrent validity was assessed by correlating the IOCv2 scales with measures from the SF-12, PostTraumatic Growth Inventory and Fear of Cancer Recurrence Inventory.

**Results:**

The French version of the IOCv2 supports the structure of the original version, with four positive impact dimensions and four negative impact dimensions. This result was suggested by the good fit of the confirmatory factor analysis and the adequate reliability revealed by Cronbach's alpha coefficients and other psychometric indices. The concurrent validity analysis revealed patterns of association between IOCv2 scale scores and other measures.

Unlike the original version, a structure with a Positive Impact domain consisting in the IOCv2 positive dimensions and a Negative Impact domain consisting in the negative ones has not been clearly evidenced in this study. The limited practical use of the conditional dimensions Employment Concerns and Relationship Concerns, whether the patient is partnered or not, did not make possible to provide evidence of validity and reliability of these dimensions as the subsets of sample to work with were not large enough. The scores of these conditional dimensions have to be used with full knowledge of the facts of this limitation of the study.

**Conclusions:**

Integrating IOCv2 into studies will contribute to evaluate the psychosocial health experience of the growing population of cancer survivors, enabling better understanding of the multi-dimensional impact of cancer.

## Background

Patients who have experienced cancer report various concerns related to the physical, psychological, social, and spiritual domains of their life [1, 2]. Large observational studies of cancer survivors have reported a range of concerns, even many years after initial treatment, including fear of recurrence, mood changes and psychological distress, concerns about body image, sexuality and fertility, and concerns about finances and employment [3–5]. The cancer experience can also provide opportunities for personal growth and strengthened relationships [6, 7].

Cancer-specific quality of life (QOL) instruments (eg: EORTC QLQ-C30 [8] and FACT-G [9]) were originally developed to assess the situation of patients during and shortly after treatment, and primarily in the context of clinical trials. These questionnaires mainly focused on acute treatment effects (nausea, fatigue, pain, insomnia) and the psychosocial effects of being diagnosed with and treated for cancer in the short term. As patients shift into the post-primary treatment survivorship phase, other issues become equally or more salient. For this reason, a number of questionnaires have been developed specifically for assessing the physical and psychosocial health experience of cancer survivors, including the Quality of Life in Adult Cancer Survivors (QLACS) [10], the Quality of Life-Cancer Survivors (QOL-CS) [11] and the Impact Of Cancer (IOC) [12]. Among them, the IOC questionnaire is the only instrument that focuses on the positive as well as the negative impacts of the disease on quality of life that long-term survivors attribute to their cancer experience.

The Impact of Cancer version 2 (IOCv2) [13] is the 47-item refined version of the IOC instrument [12]. It is organized into positive and negative impact dimensions, and several conditional dimensions applicable to subsets of survivors (see the Methods section for more details). Positive impact refers to traumatic event-related personal growth and includes lifestyle changes such as health behaviour, modification of existential values, or changes in self-evaluation as self-esteem. Negative impact relates to daily life, changes in employment and relationships, body changes. It also includes fear of cancer recurrence.

Although the IOCv2 is available in English and Dutch, it has not yet been validated for use in French-speaking populations. The current study was undertaken to provide a comprehensive assessment of the reliability and validity of the French language version of the IOCv2. The need to translate and adapt the IOCv2 into French also represents an opportunity to gain better understanding of cross-cultural differences on feelings associated with the experience of cancer, and more specifically in the phase after-treatment, such as the feeling of survivorship, largely used in Anglo-Saxon populations [14].

## Material and methods

### The IOCv2 questionnaire

The Impact of Cancer version 2 (IOCv2) is a 47-item questionnaire organized into 4 positive (altruism and empathy (AE), health awareness (HA), meaning of cancer (MOC), positive self-evaluation (PSE)) and 4 negative (appearance concerns (AC), body change concerns (BCC), life interference (LI) and worry (W)) impact dimensions [13] corresponding to the first 37 items. The questionnaire also includes 10 additional items constituting conditional dimensions applicable to subsets of survivors assessing employment concerns (EC), relationship concerns for individuals with a partner (P), and relationship concerns for those without a partner (NP). All items are scored on a five-point scale from 1 = strongly disagree to 5 = strongly agree. Each dimension score is computed as the mean of the responses for the items constituting the dimension. A higher score on a dimension implies stronger endorsement of that content area.

The original validation study of the IOCv2 in breast cancer survivors yielded high factor loadings (0.58 to 0.94), high internal consistency reliability estimates (Cronbach’s alpha coefficients between 0.78 and 0.99), and good discriminatory ability (Ferguson’s delta statistic values between 0.91 and 0.99). The negative impact dimensions scores were positively correlated with the Center for Epidemiologic Studies-Depression score. They were also positively correlated with the Breast Cancer Prevention Trial Symptom Checklist total score that assesses physical effects of medical interventions to prevent and treat breast cancer.

### Translation

The IOCv2 was originally translated into French by a project group within the European Organisation for Research and Treatment of Cancer (EORTC) Quality of Life Group using standardized, forward-backward procedures [15]. This first translation of the IOCv2 was administered in April-May 2012 to a sample of breast cancer survivors (n = 371) followed in Gustave Roussy, a large cancer center in Villejuif, France, but the psychometric analysis of this translation identified two problematic items. First, the item "I do not take my body for granted since I had cancer" had been translated to "Je ne considère plus mon corps comme quelque chose d’acquis depuis le cancer". But the analysis revealed that this item was weakly correlated with the score of its dimension Health Awareness and moderately correlated with the scores of Worry and Body Change Concerns. It was assumed that the problem lay in the translation of the idiom "take for granted". With the aim of being consistent with the concept measured through the original dimension, the item was reformulated into a statement exploring the concern about loss of confidence in the body: "Depuis le cancer je ne fais plus la même confiance à mon corps”.

Second, the item, "I consider myself to be a cancer survivor", was initially translated into "Je me considère comme un survivant du cancer", but this item was weakly correlated with its initial dimension of Positive Self-Evaluation in the French version. This item was then submitted to an internet focus group of after-cancer patients, thanks to the contribution of a dedicated cancer website, cancercontribution.fr. In French, surviving evokes mainly a feeling of loss and trauma due to a catastrophic event, and the word “survivant” in French is rather empty of pride or honor. As this item is part of the positive impact domain of IOC, we adopted a new formulation, "La traversée du cancer m'a rendue plus forte", to express that the experience of cancer makes the subject stronger. This formulation avoids focussing on negative aspects and emphasizes the positive aspect of having been confronted with cancer.

The first French translation of IOCv2 has thus been modified by integrating these two adapted items (see Appendix A). This study examines the psychometric properties of this second version of the French translation and adaptation of the IOCv2 in a breast cancer survivor sample.

### Patient sample

Female breast cancer survivors were recruited at Gustave Roussy, a large national comprehensive cancer centre, where approximately 3000 women are treated annually for breast cancer. The CANTO project including the validation study of the French translation-adaptation of the IOCv2 was approved by a research ethics committee (CPP n°11-039, Kremlin Bicetre) in October 2011. Patients attending their follow-up consultation were approached by a psychologist in April-May 2013. Patients were eligible if they were at least 18 years old, and were diagnosed with breast cancer more than 1 year earlier. Patients with mental disorders or unable to fill in the questionnaire without assistance were excluded. All consecutive eligible patients were invited to participate in the study. After providing written, informed consent, the women were asked to complete a series of questionnaires including the IOCv2, the MOS SF-12, the PostTraumatic Growth Inventory (PTGI) and the Fear of Cancer Recurrence Inventory (FCRI). Sociodemographic and clinical information were also reported by the patients. Questionnaires were completed in the waiting room of the outpatient clinic.

### Measures

The SF-12 [16, 17] is an abbreviated version of the SF-36. The information from the 12 items is summarized in physical (PCS) and emotional (MCS) component summary scores. Both scores are standardized (mean = 50, SD = 10) to the 1998 general U.S. population. A higher score indicates a better health. The high correlations between SF-12 and SF-36 summary scores (between 0.94 and 0.96 in France) support good reproduction of the SF-36 summary scores by the SF-12 [17]. The Cronbach's alpha coefficients for the physical (PCS) and emotional (MCS) component summary scores reach 0.89 showing good reliability in our sample.

The PostTraumatic Growth Inventory (PTGI) [18] measures significant positive change with 21 items and is composed of five dimensions: relating to others, new possibilities, personal strength, spiritual change and appreciation of life and a total score. All scores result from the sum of the responses of the corresponding items. A higher score on a dimension implies a higher change encountered in this domain. The Cronbach's alpha coefficients in our sample for the PTGI dimensions range from 0.69 to 0.93.

The Fear of Cancer Recurrence Inventory (FCRI) [19] is a 42-item scale assessing 7 dimensions of the fear of cancer recurrence (FCR), such as potential triggers activating FCR, the presence and severity of intrusive thoughts associated with FCR, psychological distress and functioning impairments as potential consequences of FCR, self-criticism towards FCR, and a variety of coping strategies that can be used to cope with and may influence FCR. All scores result from the sum of the responses of the corresponding items. A higher score indicates increased higher levels of FCR or of other constructs associated with the FCR. The validation study of the FCRI [19] shows good internal consistency and correlations between FCRI and three questionnaires assessing fear of cancer recurrence (between 0.68 to 0.78) and between FCRI and two questionnaires assessing psychological distress (between 0.43 to 0.66) support construct validity. The Cronbach's alpha coefficients in our sample for the FCRI dimensions range from 0.71 to 0.95.

### Statistical analysis

Confirmatory factor analysis (CFA) based on the original IOCv2 [13] 8-factor solution (reflecting the 8 hypothesized positive and negative impact dimensions that form the core of the questionnaire) was performed using covariance-based structural equation modelling with maximum likelihood estimation. Good (acceptable) fit was indicated by the following criteria: RMSEA ≤ 0.05 (0.08), SRMR ≤ 0.05 (0.10), CFI ≥ 0.97 (0.95) and NNFI ≥ 0.97 (0.95) [20]. Additional exploratory CFA’s were also conducted to evaluate the 3 conditional dimensions (employment and relationship concerns, with or without a partner) and to explore an higher-order factor structure, a positive domain consisting of the dimensions Altruism and Empathy, Health Awareness, Meaning Of Cancer and Positive Self-Evalutation and a negative domain consisting of the dimensions Appearance Concerns, Body Change Concerns, Life Interferences and Worry.

Convergent validity of each dimension was evaluated by examining the item-rest score correlations between the items and the rest scores of its hypothesized dimension (i.e. the score computed from the items of the dimension deleting that item). The convergent validity of the dimension was considered good when more than 90 % of the items of the dimension had an item-rest score correlation greater than 0.4. This indicates that the items composing the dimension are likely to be related to the same construct. Divergent validity was evaluated by examining the item-score correlations between the items and the scores of the other dimensions. The divergent validity of the dimension was considered good when more than 80 % of the items of the dimension had an item-rest score correlation with its own dimension higher than the item-score correlations with the other dimensions. This indicates that the items composing the dimension are not likely to be related to another construct.

Concurrent validity was evaluated by forming *a priori* hypotheses about patterns of association between the IOCv2 scores and the SF-12, PTGI and FCRI scores. We expected that the PTGI subscale scores would be correlated with the IOCv2 positive dimensions whereas some FCRI subscale scores (i. triggers, severity and psychological distress, and ii. functioning impairments) would be correlated with the negative ones (i. Worry, and ii. Body Change Concerns and Life Interference respectively). As the IOCv2 measures a somewhat different concept than health status, we expected only moderate associations between IOCv2 scores and SF-12 summary scores (correlation coefficient’s absolute value between 0.3 and 0.5). The correlation coefficients between dimensions of the IOCv2 were also examined. Positive dimensions (negative dimensions respectively) were expected to be highly positively correlated with one another.

Internal consistency reliability of the IOCv2 dimensions was assessed using the Cronbach's alpha coefficient [21]. Dimensions were considered reliable if α > 0.70. The ability of each dimension to discriminate among individuals was evaluated with the Ferguson's δ statistic [22, 23]. Loevinger's H scalability coefficients [24] evaluate the scalability, i.e. the quality of the scale as a unidimensional cumulative scale and the degree to which the set of items is consistent within a dimension. The scales' and items' Loevinger's coefficients (H and H_i_ respectively) were considered acceptable if H > 0.3 (high degree of homogeneity of the set of items) and H_i_ > 0.3 for all i (item consistent with the set of items). All analyses were performed with Stata 12 (Stata Corp, College Station, Texas). CFA was carried out using LISREL 8.80.

Differences in IOCv2 dimensions as a function of sociodemographic and treatment characteristics (age, time since diagnosis, treatment and partner status) were examined using Pearson correlation coefficients and Student’s *t* tests. IOCv2 scores were expected to vary across treatments, age and time since diagnosis. These additional elements of validity are to be compared with the results on the original IOCv2 validation study [13] where the correlation coefficients of the IOCv2 scores with age were weakly negative. No association was observed between the IOCv2 scores and time since diagnosis or hormonal therapy. IOCv2 scores were significantly higher for all dimensions for patients who had received chemotherapy. Finally, Altruism and Empathy, Health Awareness and Positive Self-Evaluation were scored higher for patients with a partner.

## Results

### Sample characteristics

In total, 250 women were invited to participate in the study, of whom 243 (97.2 %) agreed to do so and completed the questionnaires. Characteristics of the women are presented in Table 1. The average (standard deviation) age of the participants was 57.3 (11.3) years. Three quarters of the sample reported that they were in a relationship. Most of the subjects had a senior high school or higher level of education (78 %), and nearly half were employed during the previous 12 months. Time since diagnosis ranged from 1.1 to 35.7 years, with an average of 5.2 years. Almost all women had undergone an operation (97 %) and had received radiotherapy (95 %). The majority of women had also undergone adjuvant chemotherapy (69 %) or hormonal therapy (71 %).

**Table 1:** Subject characteristics (n = 243)

Age (years), mean ± sd (range)	57.3 ± 11.3 (30–85)
**Relationship status, nb (%)**	
Partnered	183 (76)
Not partnered	58 (24)
**Educational status, nb (%)**	
Elementary school	13 (6)
Junior high school	34 (16)
Senior high school	55 (26)
Higher education level	110 (52)
**Employment status, nb (%)**	
Employed during last 12 months	108 (45)
Not employed	134 (55)
**Time since diagnosis (years), mean ± sd (range)**	5.2 ± 4.7 (1.1-35.7)
**Chemotherapy, nb (%)**	
Yes	142 (69)
No	64 (31)
**Radiotherapy, nb (%)**	
Yes	218 (95)
No	11 (5)
**Surgery, nb (%)**	
Yes	219 (97)
No	6 (3)
**Hormone therapy, nb (%)**	
Yes	146 (71)
No	60 (29)

### Confirmatory factor analysis

Confirmatory factor analysis of the original 8-factor structure of the IOCv2 indicated a good fit suggested by most of the criteria (RMSEA = 0.045, NNFI = 0.97, CFI = 0.97) and an SRMR (=0.084) suggesting acceptable fit. Factor loadings are presented in Table 2. In addition, a subsample was constructed for each IOCv2 conditional dimension (i.e., for employment concerns, relationship concerns for those with and those without a partner), composed only of respondents for whom the conditional dimension was applicable. For each conditional dimension, an additional CFA was performed on each corresponding subsample, including the items of the conditional dimension and the 37 items of the 8 general dimensions of the IOCv2. These additional CFA’s did not yield satisfactory results for Employment Concerns and Partnered dimensions (RMSEA = 0.074, NNFI = 0.91, CFI = 0.92, SRMR = 0.11 for Employment Concerns and RMSEA = 0.069, NNFI = 0.53, CFI = 0.93, SRMR = 0.97 for Partnered). The CFA including the Not Partnered conditional dimension could not be performed due to the small number of non-partnered patients.

**Table 2:** Factor analysis results according to the original structure of IOCv2 with four negative impact domains and four positive impact domains (standardized factor loadings)

Positive impact domains		Negative impact domains	
**Health Awareness**	**Factor loadings**	**Worry**	**Factor loadings**
Item 1	0.86	Item 9	0.76
Item 2	0.87	Item 10	0.60
Item 3	0.52	Item 11	0.70
Item 4	0.53	Item 12	0.85
**Positive Self-Evaluation**		Item 13	0.79
Item 5	0.73	Item 14	0.69
Item 6	0.76	Item 15	0.83
Item 7	0.59	**Body Change Concerns**	
Item 8	0.52	Item 16	0.75
**Altruism and Empathy**		Item 17	0.77
Item 22	0.35	Item 18	0.81
Item 23	0.58	**Appearance Concerns**	
Item 24	0.79	Item 19	0.85
Item 25	0.66	Item 20	0.78
**Meaning of Cancer**		Item 21	0.66
Item 33	0.49	Life Interferences	
Item 34	0.72	Item 26	0.52
Item 35	0.81	Item 27	0.68
Item 36	0.75	Item 28	0.55
Item 37	0.80	Item 29	0.54
		Item 30	0.75
		Item 31	0.60
		Item 32	0.69

The higher-order factor structure CFA including two factors for positive domain and negative domain as well as the original 8 factors indicated an acceptable fit suggested by most of the criteria (RMSEA = 0.059, NNFI = 0.95, CFI = 0.95) except for the SRMR (=0.13).

### Score description and reliability estimates

The distribution of the scores and the psychometric indices are presented in Table 3. Scores were distributed along the range of possible values, with Ferguson's δ statistics ranging from 0.89 to 0.99, indicating good discriminating ability of all the dimensions. All dimensions but one exhibited acceptable internal consistency with Cronbach's alpha coefficients ranging from 0.71 to 0.90. The Altruism and Empathy dimension had a slightly lower coefficient (α = 0.67). The scale level Loevinger coefficients varied between 0.37 and 0.69, indicating acceptable scalability for all dimensions. The Positive impact domain was the only dimension having a scale level Loevinger coefficient smaller than 0.3 (H = 0.26). All item level Loevinger coefficients were higher than 0.3, except for item 22 of the Altruism and Empathy dimension ("I feel a special bond with people with cancer"; H_i_ = 0.25).

**Table 3:** Score description of IOCv2 dimensions and psychometric indices

IOCv2	Number of items	mean ± sd (range)	Cronbach's alpha	Ferguson's delta	Loevinger's H
**Positive impact dimensions**					
Summary Score	17	3.4 ± 0.6 (1.1-5)	0.83	0.97	0.26
Altruism and Empathy	4	3.7 ± 0.7 (1–5)	0.67	0.89	0.37
Health Awareness	4	3.8 ± 0.8 (1–5)	0.71	0.90	0.41
Meaning Of Cancer	5	2.9 ± 0.9 (1–5)	0.85	0.93	0.58
Positive Self-Evaluation	4	3.3 ± 0.9 (1–5)	0.74	0.92	0.45
**Negative impact dimensions**					
Summary Score	20	2.9 ± 0.8 (1–4.8)	0.92	0.98	0.42
Appearance Concerns	3	2.8 ± 1.2 (1–5)	0.85	0.91	0.69
Body Change Concerns	3	3.1 ± 1.2 (1–5)	0.82	0.92	0.65
Life Interference	7	2.4 ± 0.8 (1–4.7)	0.81	0.95	0.42
Worry	7	3.3 ± 1.0 (1–5)	0.90	0.96	0.62
**Employment Concerns**	3	2.4 ± 1.0 (1–5)	0.76	0.92	0.56
**Relationship Concerns (Not Partnered)**	3	2.6 ± 1.0 (1–5)	0.75	0.90	0.45
**Relationship Concerns (Partnered)**	4	1.8 ± 0.8 (1–5)	0.68	0.85	0.46

### Convergent-divergent and concurrent validity

Convergent validity was evidenced by the high percentage of item-rest score correlations higher than 0.4 (94.6 %). Most items (34/37 = 91.9 %) are more strongly correlated with their own dimension than with other dimensions, providing good evidence of divergent validity.

Table 4 shows the correlation between the scores of the IOCv2, the SF-12, the PTGI and the FCRI. The highest positive correlations within the IOCv2 are observed amongst the positive impact dimensions (Positive impact dimensions Summary score, Altruism and Empathy, Health Awareness, Meaning of Cancer and Positive Self-Evaluation) and amongst the negative impact dimensions (Negative impact dimensions Summary score, Appearance Concerns, Body Change Concerns, Life Interference and Worry). The negative impact dimensions scores are also positively correlated with the conditional dimensions (Employment Concerns, Not Partnered and Partnered).

**Table 4:** Correlation coefficients between SF-12 summary, PTGI, FCRI and IOCv2 scores

	Positive impact dimensions					Negative impact dimensions				
	SS	AE	HA	MOC	PSE	SS	AC	BCC	LI	W
**Positive impact dimensions**										
Summary score (SS)	1.00									
AE	0.72*	1.00								
HA	0.51*	0.38*	1.00							
MOC	0.80*	0.39*	0.10	1.00						
PSE	0.73*	0.35*	0.12	0.50*	1.00					
**Negative impact dimensions**										
Summary score (SS)	0.09	0.29	0.48*	−0.16	−0.18	1.00				
AC	−0.06	0.03	0.22	−0.18	−0.15	0.68*	1.00			
BCC	0.06	0.22	0.35*	−0.12	−0.16	0.83*	0.61*	1.00		
LI	0.14	0.35*	0.37*	−0.09	0.09	0.87*	0.49*	0.67*	1.00	
W	0.10	0.26	0.51*	−0.15	−0.19	0.84*	0.37*	0.55*	0.60*	1.00
**EC (N = 106)**	0.09	0.25	0.26	−0.04	−0.13	0.45*	0.19	0.31*	0.53*	0.34*
**NP (N = 57)**	−0.13	0.21	0.26	−0.33*	−0.16	0.60*	0.49*	0.34*	0.63*	0.51*
**P (N = 168)**	0.14	0.22	0.17	0.02	0.05	0.42*	0.26	0.18	0.51*	0.32*
**SF-12 MCS**	0.03	−0.16	−0.20	0.16	0.16	−0.61*	−0.36*	−0.46*	−0.56*	−0.53*
**SF-12 PCS**	−0.07	−0.14	−0.12	0.07	−0.06	−0.40*	−0.25	−0.55*	−0.35*	−0.24
**PTGI**										
Relating to others	0.62*	0.51*	0.28	0.53*	0.38*	−0.02	−0.07	−0.01	−0.03	0.01
New possibilities	0.64*	0.39*	0.18	0.69*	0.41*	−0.10	−0.11	−0.14	−0.03	−0.08
Personal strength	0.63*	0.35*	0.11	0.64*	0.56*	−0.18	−0.12	−0.12	−0.16	−0.16
Spiritual change	0.45*	0.35*	0.14	0.41*	0.31*	−0.04	−0.06	−0.05	0.02	−0.06
Appreciation of life	0.58*	0.36*	0.32*	0.55*	0.32*	0.02	−0.11	−0.02	0.05	0.06
Total score	0.73*	0.50*	0.26	0.70*	0.49*	−0.08	−0.11	−0.08	−0.04	−0.06
**FCRI**										
Triggers	0.17	0.32*	0.37*	−0.06	−0.01	0.45*	0.18	0.18	0.36*	0.56*
Severity	0.11	0.28	0.45*	−0.16	−0.08	0.64*	0.31*	0.37*	0.47*	0.74*
Psychological distress	0.15	0.31*	0.38*	−0.08	−0.06	0.55*	0.28	0.28	0.46*	0.60*
Functioning impairments	0.08	0.27	0.28	−0.09	−0.12	0.66*	0.34*	0.44*	0.64*	0.59*
Insight	0.19	0.28	0.26	0.00	0.06	0.40*	0.15	0.18	0.42*	0.40*
Reassurance	0.20	0.24	0.27	0.05	0.07	0.32*	0.11	0.20	0.26	0.35*
Coping strategies	0.30*	0.33*	0.30*	0.14	0.14	0.20	0.03	0.12	0.14	0.26
Total score	0.23	0.40*	0.47*	−0.05	−0.01	0.64*	0.28	0.35*	0.54*	0.70*

* indicates correlation coefficient ≤ −0.30 or correlation coefficient ≥0.30

With regard to the correlations with other instruments, the scores of the Altruism and Empathy, Meaning Of Cancer and Positive Self-Evaluation dimensions were positively correlated with all the PTGI scores. High scores on PTGI scales indicate more posttraumatic growth, whereas high scores on IOCv2 positive dimensions reflect more positive impacts of cancer. In contrast, the negative dimensions Life Interference and Worry of the IOCv2 showed positive correlations with all the dimensions of the FCRI except Coping Strategies. High scores on the FCRI dimensions indicate more concerns about cancer recurrence and high scores on the IOCv2 negative dimensions reflect more negative impacts of cancer. All the negative dimensions of the IOCv2 (Appearance Concerns, Body Change Concerns, Life Interference and Worry) were negatively correlated with the Mental Component Summary score of the SF-12, and the Body Change Concerns and Life Interference dimensions of the IOCv2 also correlated negatively with the Physical Component Summary score of the SF-12. High scores on the SF12 indicate better health status, and are associated with lower levels of negative impacts as assessed by the IOCv2.

### Additional elements for validity

The associations observed between the IOCv2 dimensions and sociodemographic and medical variables are presented in Table 5. The very weak correlations of the IOCv2 dimensions scores with time since diagnosis indicate that the impact of the cancer experience, both positive and negative, did not change significantly as a function of time. Age was most strongly and negatively correlated with The Health Awareness dimension of the IOCv2. Younger women scored higher than older women, indicating a greater awareness of health. Respondents who received chemotherapy tended to score higher than others on both positive and negative impact dimensions. In particular, those women who had undergone chemotherapy were more likely to report more concerns about appearance. All these trends are in accordance with the original IOCv2 validation study [13]. Women who had undergone radiotherapy tended to have higher scores on the negative impact dimensions of the IOCv2 and lower scores on the positive impact dimensions, especially for the Meaning of Cancer dimension, than women who had not undergone radiotherapy. Women who received hormonal therapy tended to score higher than others on negative impact dimensions of the IOCv2. No patterns emerged for women who had surgery probably because only 6 women (3 %) did not have surgery. Patients without partner were more likely to report relationship concerns when they had not undergone hormonal therapy than patients without partner that had undergone hormonal therapy.

**Table 5:** Correlations between IOCv2 dimensions and sociodemographic and treatment characteristics and mean scores of IOCv2 dimensions

			Chemotherapy		Radiotherapy		Surgery		Hormonotherapy		Partnered	
	Time since diagnosis	Age	No	Yes	No	Yes	No	Yes	No	Yes	No	Yes
			N = 64	N = 142	N = 11	N = 218	N = 6	N = 219	N = 60	N = 146	N = 58	N = 183
**Positive impact dimensions**												
SS	−0.05	−0.13*	3.28	3.43	3.65	3.37	3.42	3.39	3.40	3.42	3.37	3.47
AE	0.01	−0.08	3.56	3.73	3.68	3.68	3.63	3.70	3.72	3.71	3.71	3.68
HA	−0.08	−0.17*	3.75	3.86	4.00	3.80	4.00	3.81	3.80	3.88	3.87	3.80
MOC	0.01	−0.02	2.74	2.90	3.45*	2.83*	2.77	2.86	2.89	2.91	2.99	2.84
PSE	−0.08	−0.11	3.21	3.38	3.52	3.32	3.50	3.34	3.36	3.32	3.42	3.31
**Negative impact dimensions**												
SS	−0.04	−0.04	2.70	2.92	2.66	2.85	3.12	2.85	2.77	2.91	2.85	2.91
AC	−0.03	−0.12	2.44*	2.85*	2.73	2.70	2.93	2.71	2.42*	2.82*	2.70	2.78
BCC	−0.05	−0.01	2.93	3.18	2.61	3.08	3.17	3.08	2.9	3.18	3.05	3.09
LI	−0.01	0.00	2.30	2.47	2.17	2.43	2.71	2.42	2.39	2.47	2.54	2.38
W	−0.04	−0.03	3.11	3.31	3.14	3.25	3.54	3.25	3.25	3.29	3.31	3.25
			N = 28	N = 73	N = 5	N = 100	N = 3	N = 101	N = 27	N = 71	N = 79	N = 27
**EC**	−0.06	0.07	2.21	2.48	2.27	2.43	2.56	2.42	2.53	2.34	2.40	2.44
			N = 11	N = 40	N = 2	N = 53	N = 1	N = 53	N = 10	N = 42		
**NP**	−0.14	0.03	2.33	2.75	2.50	2.63	NA	NA	3.33*	2.42*		
			N = 51	N = 95	N = 9	N = 155	N = 4	N = 158	N = 45	N = 103		
**P**	0.13	0.19*	1.77	1.78	1.33*	1.82*	2.38	1.77	1.63	1.85		

* p value of the *t* test < 0.05

NA: not applicable

## Discussion

In this paper, we have reported the results of a study of the psychometric performance of the French language-version of the IOCv2 questionnaire. The good fit of the confirmatory factor analysis suggests that the French version supports the 8-factor structure of the original version, with four positive impact dimensions and four negative impact dimensions. The Cronbach's alpha coefficients and other psychometric indices indicate adequate reliability, and the correlation coefficient values support the validity of the French version. As expected, we have found negative correlations between some of the negative dimensions of the IOCv2 and the Mental and Physical Component scores of the SF12. We also observed positive correlations between the positive dimensions of the IOCv2 and the PTGI scores, and between the negative dimensions of the IOCv2 and the FCRI scores. These results support the construct validity of the IOCv2, and its ability to capture a range of positive and negative psychosocial health experiences with a relatively limited number of items.

Nevertheless, we note weak correlation coefficients between some positive dimensions, in particular between HA and MOC (0.10), and HA and PSE (0.12). These results point out the fact that the four positive dimensions do not form a consistent set of items. Consequently, the construct of a summary score by aggregating these four positive dimensions produces a set of items with a poor scalability (H = 0.26). This result could explain medium performance of the hierarchical CFA. A structure with a Positive Impact domain consisting in the IOCv2 positive dimensions and a Negative Impact domain consisting in the negative ones has not been clearly evidenced in this study.

The limited practical use of the conditional dimensions Employment Concerns and Relationship Concerns, whether the patient is partnered or not, did not make possible to provide evidence of validity and reliability of these dimensions as the subsets of sample to work with were not large enough. The scores of these conditional dimensions have to be used with full knowledge of the facts of this limitation of the study.

A low correlation has been observed between the item ‘I feel a special bond with people with cancer’ and the score computed using the other items of its dimension Altruism and Empathy. This might be explained, at least in part, by cultural differences in the attitudes in the way that survivors feel connected (or not) with the cancer survivor community. In contrast to some countries, it is our impression that engagement in community and advocacy movements is low among French-speaking survivorship population, thus resulting in a limited sense of group identity. This result is in accordance with the results of the comparison between Dutch and American non-Hodgkin lymphoma survivors regarding IOC [25] where the IOC questionnaire is said to be culturally sensitive. The paper pointed out that the cultural differences between USA and Western Europe in terms of social safety net and health care systems as well as the well-developed programs in cancer survivorship care such as social support in North America might explain that the concepts of altruism and empathy and the feeling of belonging to a group of cancer survivors are more developed in USA.

It should be mentioned here that reformulation of items 1 and 5, as explained in the Methods section, has allowed better convergence between the original version and its translation. Therefore, we can consider that results drawn from both versions of the IOCv2 through two language-wise different samples may be equally reliable and allow transcultural comparisons.

Sociodemographic characteristics of our sample differed from those of the original U.S. validation study. French-speaking breast cancer survivors were younger, more often employed and more often partnered. These are characteristics that may contribute to better psychosocial adjustment during the survivorship period. There were also differences between the two study samples in disease and treatment-related factors. French-speaking women responded to the questionnaire less than 5 years after diagnosis, and therefore they were not considered yet as long-term survivors. Moreover, a greater percentage of the French-speaking sample had undergone chemotherapy, treatment associated with significant long-term toxicity effects. Yet, despite these differences in sample composition, the psychometric performance of the IOCv2 was consistently good.

Our study has several limitations that should be mentioned. First, the study was restricted to breast cancer survivors which implies that further validation work across different cancer diagnoses is needed to propose this tool as a widely useful instrument for cancer survivors. Evidence from the U.S. [26, 27] and the Netherlands [25, 28] suggest that the IOCv2 is also suitable for use with other diagnostic groups. Second, the study employed a cross-sectional design. Future longitudinal studies are needed to investigate the responsiveness of the IOCv2 to changes over time in the survivorship experience. This is particularly important, because the data from our cross-sectional investigation such that the psychosocial impact of cancer and its treatment during the post-primary treatment survivorship period remains relatively stable. Only a longitudinal study design can provide the evidence needed to determine if this is in fact the case. Finally, and conversely, well-designed studies are needed to examine the stability of the IOCv2; that is, its reproducibility under conditions of no or limited change.

Our study also had a number of strengths. First, our psychometric study was based on a thorough and carefully conducted translation process that ensured that the French language version of the IOCv2 to be tested was of high quality. Second, the study sample was relatively large and has a very high participation rate, supporting the generalizability of our findings to the population of French breast cancer survivors. Finally, we premised much of the analysis (e.g., the confirmatory factor analysis and the validity testing) on *a priori* hypotheses about the structure of the questionnaire and its association with other measures and with various sociodemographic and clinical variables.

The French-language version of the IOCv2 is currently being used as one of the assessment tools of a recently created, large cohort of breast cancer survivors in France, the CANTO cohort. Creation of this cohort, with 12,000 women with non-metastatic breast cancers, will facilitate the study of long term physical, psychological, social, and economic consequences of the primary breast cancer and its treatment. Importantly, use of the IOCv2 in this study will bring not only the negative, but also the positive consequences of having had breast cancer into focus. The IOCv2 will allow such a detailed evaluation through a limited number of items. As the IOCv2 focuses on quality of life impacts attributed to the cancer experience, it remains recommended to jointly use other generic HRQOL measures to evaluate other health related quality of life aspects, as applied in the assessment of the CANTO cohort that integrates the current validation work. The information generated by this and future epidemiological and clinical studies will hopefully contribute to developing optimal clinical pathways and survivorship care plans for women with breast cancer.

## Conclusion

The IOC questionnaire was originally developed and validated among a mixed sample of cancer survivors. The IOCv2 questionnaire, a refined version of the IOC, was validated among long-term breast cancer survivors in the United States. The impetus for developing the questionnaire was an awareness of the need to evaluate the psychosocial health experience of the growing population of cancer survivors, enabling better understanding of the multi-dimensional impact of cancer. The French version of the IOCv2 demonstrates good psychometric properties, and thus is appropriate for use in studies in France and perhaps in other French-speaking countries as well (although this latter use needs to be confirmed).

Results from studies using the IOCv2 may contribute not only to enhance descriptive research on cancer survivors’ unmet psychosocial needs, but also to designing high quality research on the effectiveness of psychosocial interventions aimed at improving the cancer survivorship experience.

## Appendix A: French version of the IOCv2 questionnaire

### Questionnaire sur l’impact du cancer, IOC version 2

**Table 6:** Instructions: étant donné votre vie actuelle, que ressentez-vous par rapport au fait d’avoir eu un cancer ? *Veuillez entourer le chiffre décrivant le mieux à quel point vous êtes d’accord ou non avec chacun des énoncés*

		Pas du tout d’accord	Pas d’accord	Neutre	D’accord	Tout à fait d’accord
		1	2	3	4	5
1.	Depuis le cancer je ne fais plus la même confiance à mon corps					
2.	Je suis plus soucieux(se) de ma santé depuis que j’ai eu un cancer					
3.	Je suis plus conscient(e) des problèmes physiques ou des changements de mon corps depuis que j’ai eu un cancer					
4.	Je prends mieux soin de moi (de ma santé) depuis que j’ai eu un cancer					
5.	La traversée du cancer m’a rendu plus forte					
6.	Je ressens un sentiment de fierté ou d'accomplissement pour avoir survécu au cancer					
7.	Avec le cancer, j’ai appris quelque chose sur moi-même					
8.	J’ai l’impression d’être un modèle pour les autres personnes atteintes d’un cancer					
9.	Je doute de mon avenir depuis que j’ai eu un cancer					
10.	J’ai l’impression qu’il ne me reste plus beaucoup de temps à vivre					
11.	Je m’inquiète à l’idée que le cancer revienne ou d’avoir un autre cancer					
12.	Je doute de ma santé depuis que j’ai eu un cancer					
13.	Je m’inquiète au sujet de mon avenir					
14.	Lorsque j’ai de nouveaux symptômes (douleurs, tomber malade ou attraper la grippe), je m'inquiète d’un retour du cancer					
15.	Je m'inquiète au sujet de ma santé					
16.	Je suis préoccupé(e) par le fait de ne pas avoir retrouvé l’énergie que j’avais avant d’avoir un cancer					
17.	Je suis gêné(e) par le fait que mon corps ne puisse plus faire ce qu’il faisait avant d’avoir eu le cancer					
18.	Je me sens vieux/vieille depuis que j’ai eu un cancer					
19.	Je m’inquiète au sujet de l’apparence de mon corps					
20.	Je me sens défiguré(e)					
21.	Je couvre parfois les parties de mon corps que je ne veux pas que les autres voient					
22.	J’ai l’impression d’avoir un lien particulier avec les personnes atteintes d’un cancer					
23.	Du fait que j’ai eu un cancer, je comprends mieux ce que peuvent ressentir les autres quand ils sont gravement malades					
24.	J’ai davantage envie d’aider les autres depuis que j’ai eu le cancer					
25.	J’ai l’impression que je devrais donner quelque chose en retour aux autres du fait que j’ai survécu au cancer					
26.	Je culpabilise aujourd'hui de ne pas avoir été disponible pour ma famille lorsque j'avais le cancer					
27.	J’ai l’impression que le cancer dirige ma vie					
28.	Je me sens seul(e) depuis que j’ai eu un cancer					
29.	Depuis que j’ai eu un cancer, j’ai l’impression que certaines personnes (amis, famille, collègues) ne me comprennent pas					
30.	L’incertitude de mon avenir influence mes décisions de faire des projets (par exemple: travail, loisirs/voyages, se marier, s’engager dans une relation, avoir une famille, aller en cours)					
31.	Je ne peux plus faire les activités que j’aime (par exemple: voyager, avoir une vie sociale, des loisirs, passer du temps en famille) depuis que j’ai eu un cancer					
32.	Les symptômes actuels liés au cancer ou au traitement (par exemple: problèmes de contrôle de la vessie ou des intestins, lymphœdème, chute des cheveux, cicatrices, stérilité, ménopause précoce, manque d’énergie, impuissance/problèmes sexuels, douleurs ou gêne physique) perturbent ma vie					
33.	Le fait d’avoir eu un cancer m’a donné une raison de faire des changements dans ma vie					
34.	Suite au cancer, j’arrive mieux à exprimer ce que je veux					
35.	Suite au cancer, j'ai davantage confiance en moi					
36.	Le fait d’avoir eu un cancer a donné un sens à ma vie					
37.	J’ai l’impression de mieux contrôler ma vie depuis que j’ai eu un cancer					
